# ESCAIDE 2025: call for abstracts

**DOI:** 10.2807/1560-7917.ES.2025.30.15.250417m

**Published:** 2025-04-17

**Authors:** Eurosurveillance editorial team

**Affiliations:** 1 European Centre for Disease Prevention and Control (ECDC), Stockholm, Sweden

The European Scientific Conference on Applied Infectious Disease Epidemiology (ESCAIDE) 2025 will be organised as a hybrid event, in Warsaw, Poland, and online, from 19 to 21 November 2025. 

The call for abstracts for this year’s edition of ESCAIDE, the annual public health conference organised by the European Centre for Disease Prevention and Control (ECDC), is now open. Abstracts in all areas applied to infectious disease and public health are welcomed. **The deadline for submissions is 14 May.**

Learn more and submit your abstract.

